# Case Report: Delayed presentation of penile epidermoid cyst following reconstruction for Peyronie’s disease

**DOI:** 10.12688/f1000research.7232.1

**Published:** 2015-11-24

**Authors:** Luriel I. Smith-Harrison, Jacques Farhi, Raymond A. Costabile, Ryan P. Smith

**Affiliations:** 1 Department of Urology, University of Virginia Health System, Charlottesville, VA, 22908, USA

**Keywords:** Penile mass, Epidermoid cyst, Peyronie’s Disease

## Abstract

Penile masses are a concerning finding for both patient and clinician upon initial presentation. There is a wide differential for penile masses from the benign (fibrous plaques, cysts, ulcerative lesions, benign penile pearly papules, etc.) to more concerning malignant lesions. A proper history and physical is the first step to determining the etiology of the mass and any future clinical interventions. In this paper, we review a case of a 73-year-old male who is found to have an enlarging mass during work-up for possible placement of inflatable penile prosthesis. Fortunately, the mass was determined to be a benign epidermoid cyst presenting thirty years after reconstruction for Peyronie’s disease using dermal penile skin graft. With this unique presentation we review the scant literature on penile mass formation following Peyronie’s repair.

## Introduction

Peyronie’s disease is a common urologic entity with multiple options for definitive surgical repair. Plaque excision with grafting is a known and accepted method for reconstruction. There are multiple options for graft material, each carrying its own specific risk for complications and comorbidities ^1^. In this particular case, we discuss Peyronie’s disease treated with plaque excision and dermal skin grafting.

## Case presentation

A 73-year-old man was referred to our clinic in surgical consultation for possible placement of inflatable penile prosthesis due to progressively worsening erectile dysfunction. At his initial visit, he was found to have a non-tender rapidly growing mass in the distal penile shaft, which prohibited him from using his vacuum erection device. His past medical history was significant for type 2 diabetes and Peyronie’s disease. Thirty-two years prior, he underwent corrective surgery for Peyronie’s disease. Operative and clinical notes from that period could not be obtained, though the patient reported the procedure included plaque excision and use of a dermal penile skin graft. Following the procedure, he only reported mild residual penile desensitization.

On exam, we noted a well-healed surgical scar and a 3 cm nodule arising from the left lateral aspect of the distal shaft. Moderate corporal fibrosis was also noted. We did not appreciate any concerning erythema, tenderness, drainage or ulceration. Importantly, the size and location of the mass prevented the patient from using a vacuum erection device. With the atypical presentation of this mass, the decision was made to proceed with further work-up prior to discussing any interventions for his erectile dysfunction.

**Figure 1.**
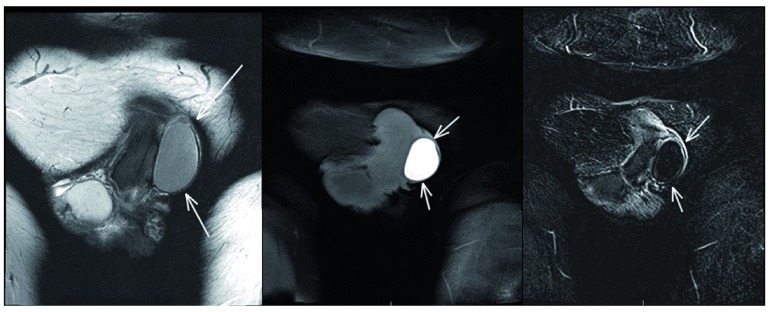
**a**: T-2 MRI reveals non-enhancing 4.1 cm lesion abutting the left corpus cavernosa and exerting mild compression on the left corpus cavernosum. **b**: T-1 MRI without contrast shows a homogenous rim enhancing lesion without solid components. **c**: Subtraction MRI of lesion. Differential for this lesion based on imaging is proteinaceous fluid versus subacute hemorrhage.

From this point, we proceeded with pelvic magnetic resonance imaging (MRI) without contrast ( Figure 1). This revealed a 2.4 × 2.8 × 4.1 cm, rim-enhancing, hemorrhagic mass without internal solid components. The mass was abutting and mildly compressing the left corpus cavernosum. The mass did not invade the corpus cavernosum and the tunica albuginea was intact, though thickened. The right corpus cavernosum and corpus spongiosum were normal.

Penile duplex ultrasound, performed after an injection of 10 mcg alprostadil into the right corpora, revealed a 4 cm mass with complex internal echoes without Doppler flow. Compression of the corpora was seen with a moderate wasting deformity opposite of the mass. Approximately 15 degrees of mild leftward deviation was noted. Arterial peak flow was estimated at 12 cm/sec and resistive indices were 0.6 bilaterally. In addition, plaque without calcification was seen in the mid-shaft.

Given the constellation of residual penile curvature, erectile dysfunction which was non-responsive to phosphodiesterase inhibitors, and the presence of a penile mass, the patient elected for placement of a three-piece inflatable penile prosthesis in conjunction with excision of the mass. We reviewed the possibility of other adjunct procedures such as penile modeling, grafting and plication. Penile prosthesis placement was declined by the patient’s insurance and mass excision was pursued alone. An incision was made over the site of the mass which was removed in its entirety without complication. During dissection, previous sutures from the dermal graft were appreciated. The corporal body was left intact. The pathology report was consistent with a benign inclusion cyst and his post-operative recovery was unremarkable with discharge to home immediately following surgery. Upon close follow-up, his penile curvature is stable, as determined by clinical exam and he has resumed using a combination of phosphodiesterase inhibitors and a vacuum erection device.

## Discussion

There are multiple options for surgical management of Peyronie’s disease. Part of the treatment algorithm includes a number of options for grafting. It is well-known that skin grafts carry a greater risk of transplanting apocrine glands and hair follicles to the donor site. Due to this, it is incumbent upon the surgeon to pick a graft best suited for the operative site and graft intent. The surgeon must also weigh the risks and benefits of each possible donor site. Although this principle is followed in reconstructive surgery for Peyronie’s disease, there is a paucity of case reports documenting cyst formation after dermal graft inlay procedures. The authors most commonly use small intestinal submucosa or tunica vaginalis grafts.

To our knowledge, there are two case reports describing this complication ^2, 3^. One case report describes a middle-aged male who presented with a unilateral enlarging penile lesion 2 years after having a dermal graft procedure for Peyronie’s disease. Upon exploration, a fluid filled keratin mass containing hair was removed ^2^. The other case report described an elderly man who had dermal graft repair for a dorsal plaque ^3^. The graft was harvested from a site devoid of hair, the abdominal wall near the left flank. Four years later the patient developed a swelling at the dorsum of the penis. In these two cases, the cyst presented less than 5 years after the operation.

In our case, the inclusion cyst presented more than 30 years after the operation, suggesting that cyst formation can be sporadic and yet rapid. The latency of cyst formation could be due to more extensive de-epithelialization of the graft in our case compared to the other cases. Still rapidly expanding soft tissue penile masses could be concerning for a neoplasm, albeit extremely rare. Therefore rapidly expanding soft tissue penile masses should be investigated with MRI to rule out a neoplasm and to further classify the lesion and location, which could prove to be valuable in surgical planning ^4, 5^. However, Peyronie’s disease is not thought to be a predisposition to a penile neoplastic lesion and to date the literature is devoid of a penile neoplasm after a dermal graft procedure ^6^.

## Conclusions

The development of any penile mass should be concerning and warrants a full work-up by the appropriate medical provider. As this case shows, benign epidermoid cysts must be considered in those patients with a history of prior skin graft to the penis. Options for management of erectile dysfunction should not be limited following excision of an epidermoid cyst.

## Consent

Written, informed consent for publication of clinical details and images was sought and obtained from the patient.
