# Supplementary material for: Case Report: Delayed presentation of penile epidermoid cyst following reconstruction for Peyronie’s disease
Source: F1000Res. 2015 Nov 24;4:1337. [Version 1] doi: 10.12688/f1000research.7232.1 (PMC4706047; doi:10.12688/f1000research.7232.1)
Supplement: Peer review history

# Peer review: Referee response for version 1

**DOI:** 10.5256/f1000research.7792.r11314

**Published:** 2015-12-14

**Authors:** Alexander W. Pastuszak

**Affiliations:** 1 Scott Department of Urology, Division of Male Reproductive Medicine and Surgery, Baylor College of Medicine, Houston, TX, USA

The authors provide a much needed perspective on penile masses in the setting of Peyronie's disease after surgical repair. The manuscript is well written and appropriately identifies an overall paucity of studies examining this complication, summarizing the extent of our knowledge in these specific situations in a total of 2 other available case reports. As such, this work is a very useful and important contribution to the literature. There are two points on which additional information would be useful, however. First, with regards to the patient case, it would be useful to include any serum studies that were performed, which may be salient particularly in the setting of a mass that is not benign. Second, a short discussion of the rarity (i.e. incidence / prevalence) of malignant penile masses in this type of setting would be helpful.

I have read this submission. I believe that I have an appropriate level of expertise to confirm that it is of an acceptable scientific standard.

# Peer review: Referee response for version 1

**DOI:** 10.5256/f1000research.7792.r11315

**Published:** 2015-11-30

**Authors:** James M. Hotaling

**Affiliations:** 1 Division of Urology, Department of Surgery, Center for Reconstructive Urology and Men's Health, University of Utah, Salt Lake City, UT, USA

This is a well done article on a rare entity that urologists might encounter once or twice in a career.  Penile masses are certainly challenging and there is little data on their management.

I have read this submission. I believe that I have an appropriate level of expertise to confirm that it is of an acceptable scientific standard.
